# A Noninvasive Neuroprosthesis Augments Hand Grasp Force in Individuals with Cervical Spinal Cord Injury: The Functional and Therapeutic Effects

**DOI:** 10.1155/2013/836959

**Published:** 2013-12-30

**Authors:** Rune Thorsen, Davide Dalla Costa, Sara Chiaramonte, Luca Binda, Ettore Beghi, Tiziana Redaelli, Eugenio Occhi, Maurizio Ferrarin

**Affiliations:** ^1^IRCCS-Fondazione Don Carlo Gnocchi, Via Capecelatro 66, 20148 Milan, Italy; ^2^Spinal Unit, Ospedale Niguarda Ca' Granda, Piazza dell'Ospedale Maggiore 3, 20162 Milan, Italy; ^3^Spinal Unit, Ospedale Morelli, Via A. Zubiani 33, Sondalo, 23035 Sondrio, Italy; ^4^Laboratory of Neurological Disorders, Istituto Mario Negri, Via La Masa 19, 20156 Milan, Italy

## Abstract

*Objectives*. The primary purpose of this study was to evaluate myoelectrically controlled functional electrical stimulation (MeCFES) for enhancing the tenodesis grip in people with tetraplegia. The second aim was to estimate the potential number of candidates for the MeCFES device. The application of MeCFES provides the user with direct control of the grasp force as opposed to triggered FES systems. *Methods*. Screening 253 medical records of C5 to C7 spinal cord injury resulted in 27 participants who trained activities of daily living for 12 × 2 hours, using the MeCFES. Hand function was evaluated by the Action Research Arm Test (ARAT). Primary outcome was the ARAT change score with/without the device, before/after the intervention period. Secondary outcome was the number of positive or clinically relevant change scores with respect to the cohort. *Results*. The MeCFES improved hand test score in 63% of the subjects at first application. Training resulted in a significant therapeutic effect, which resulted in an overall increase of hand function in 89% of the participants and 30% experienced a clinically relevant change (6 points or more). *Conclusions*. Clinical relevance was found both as an assistive aid and as a therapeutic tool in rehabilitation. The therapeutic effect deserves further investigation in clinical studies.

## 1. Introduction

The majority of cervical spinal cord injuries fall into the segment of C5–C7 [1]. Such lesions will spare proximal control though severely impairing the hand function. Improvement of the hand function is highly prioritized among tetraplegic individuals [2] due to its impact on personal independence [3]. Early after the lesion, conservative management of the hand aims to promote the tenodesis grip [4]. If the passive mechanical tenodesis properties are adequate, this grip is used as follows: with the wrist in flexion, the fingers are manipulated around the target. Then the wrist is actively extended, causing passive finger flexion and the target can then be held. The effectiveness of the tenodesis for activities of daily living (ADL) depends in part on the resulting grasp force [4]. Reconstructive surgery [5] or implanted functional electrical stimulation (FES) devices [6] may improve grasp [7]. Yet, some patients may refrain from surgery [2]. Surface stimulation systems often precede implanted systems, since they can be applied without commitment and safety issues and have demonstrated their validity as an assistive technology for daily use [2, 8, 9].

FES systems are typically controlled by shoulder movements [6] or triggered to activate a fixed stimulation sequence [8, 9]. Though direct myoelectric control has the advantage of being cognitively simpler to use, it has received less attention [10–12]. As for the expected number of users, it has been estimated that 60% of the tetraplegic population could benefit from reconstructive surgery [5] and 7% could be candidates for an implanted FES system [13]. It is generally estimated that nearly half the population with a C5 to C7 neurological level would be interested in such assistive technologies [2, 8, 9, 13].

The effect of training ADL in occupational therapy is assumed to be beneficial and is a key element in the rehabilitation process [14]. Though many aspects besides function must be taken into account when evaluating assistive technologies [15], performance testing of hand function is central.

Upper limb evaluation tools, specific for tetraplegia, are used in the scientific literature (e.g., the Sollerman hand function test [16] and the grasp-and-release test [17]) and still more are being proposed (e.g., the AuSpinal [18], the Van Lieshout test [19], and the GRASSP [20]). Recently, also the more general Action Research Arm Test (ARAT) [21] has been used in studies targeting the tetraplegic hand [22].

A previous study on myoelectrically controlled FES (MeCFES) demonstrated that active wrist extension could be used to control the stimulation of the finger flexors, so as to enhance the tenodesis grip. Specifically, the active muscles were in direct control of the stimulation intensity and, as a consequence, an increase of wrist extension was producing increasingly more finger flexion. In a 20-subject convenience sample, 5 hands gained an immediate improvement of the system with respect to nonuse of the system [12].

The purpose of this research paper is to quantify how much hand function can be improved with the MeCFES device both immediately and in the longer terms and to estimate the proportion of subjects with tetraplegia who would benefit from this method.

## 2. Methods

**Figure 1:**
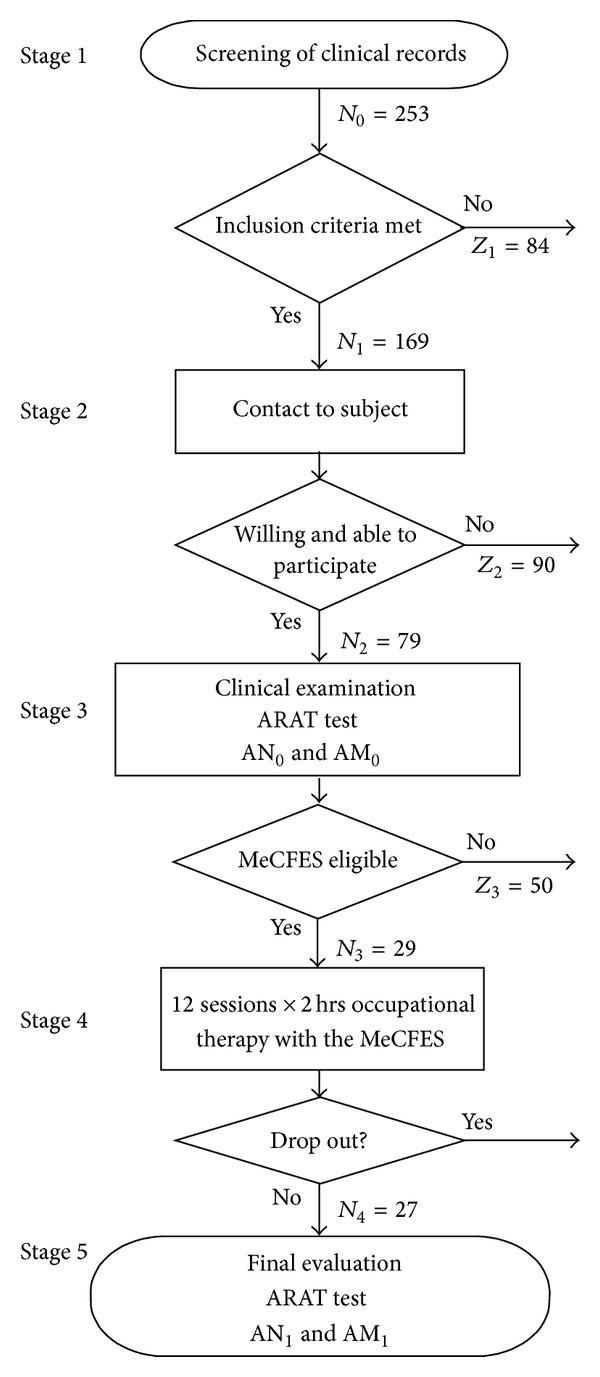
Flowchart of the study. The outcome measures were the ARAT test scores obtained in two times and two conditions: natural (AN) and with the MeCFES (AM), before (subscript zero) and after (subscript one) the training period.

We conducted this study at the two largest spinal units in northern Italy. The study had the following five stages: (1) screening a cohort of clinical records to register cases of cervical spinal cord lesion and identify candidates for the neuroprosthesis; (2) asking candidates to participate in a (3) clinical evaluation of the hand function; (4) inviting subjects fulfilling inclusion criteria to participate in an intervention period of occupational therapy; (5) a final evaluation of the hand function (see flowchart of the study Figure 1). The purpose was to estimate how many subjects would participate in the study and what fraction of them resulted candidates for the neuroprosthesis and how much they would gain in hand function.

### 2.1. The Neuroprosthesis

The neuroprosthesis was a one-channel battery powered portable research prototype implementing myoelectrically controlled functional electrical stimulation (MeCFES) [12]. The myoelectric signal was processed such that the stimulation amplitude would be a smoothened estimate of the muscle activity. Stimulation was a biphasic 300 *μ*s current pulse with 16 pulses per second. The stimulation amplitude was calculated as a gain times the offset compensated estimate of volitional myoelectric activity. An upper limit was applied to avoid excessive stimulation [23, 24]. Standard surface self-adhesive stimulation electrodes (Axelgaard, Fallbrook, USA) and EMG recording electrodes (Kendall Arbo; Covidien, Germany) were used and connected to the MeCFES unit by flexible cables. During exercises this was put in a pocket of the patient or mounted on the wheelchair. Electrode positions were identified for each patient by trial-starting with recording electrodes over the muscle belly of extensor carpi radialis/ulnaris and stimulation electrodes over the flexor digitorum superficialis and/or flexor polices longus and then modified to optimise control and provide functional finger and/or thumb flexion. The system was calibrated for each patient by a laptop connected to the device. Parameters were iteratively adjusted by the clinician to obtain direct control of finger flexion as being directly related to the amount of wrist extension. Thus, the user would have direct control of the stimulation intensity by the degree of volitional wrist extension in synergy, adding force to the tenodesis grasp [12].

### 2.2. Outcome Measurement

The ARAT presents the best trade-off between time to administer and validity/reliability regarding the intervention [22]. To minimize muscle fatigue and burden on the patient, it was chosen to include no other performance evaluations [25]. This test has been validated [26] against the Fugl-Meyer assessment [27], the Sollerman test (*r* = 0.94) [28], and other common hand function tests [27, 29]. It is categorized with grasp, grip, pinch, and gross movement; lifting and moving blocks of various sizes, pouring water, picking up, and placing small objects; and gross movement of hand to mouth, head, and neck. It measures the performance of specific tasks on a scale from 0 to 57 under standardised conditions. A positive change score exceeding 5.7 is considered clinically relevant [26].

### 2.3. Recruitment of Participants

First, the databases of the spinal units were screened to select records of cervical spinal cord lesions. Records of (re)admission within an eight-year period were collected. In the second stage, subjects with C5 to C7 neurological level of lesion, time since onset in excess of 6 months, and age between 18 and 80 years were contacted. Exclusion criteria were presence of implanted devices, cardiovascular problems, epilepsy, peripheral lesion of the brachial plexus, and respiratory, renal, cardiac, and cognitive and aphasic problems. Contacted subjects were furthermore inquired for their hand status and excluded if no hand was functional or if both hands were fully functional. In stage 3, subjects willing and able to participate were seen in the clinic to verify if they had wrist extension above grade 1 on the MRC scale and innervation of the finger flexors, that is, motor response to stimulation of the finger and/or thumb flexors. Subjects were excluded if none of the hands were functional. Nonfunctional hands were characterized by adverse spasticity, contractures, deformities, or important range of movement limitations of the joints. Subjects with both hands fully functional (i.e., completing all tasks in the ARAT) were also excluded. Motor response was tested with a stimulation intensity of maximal tolerable level up to 60 mA. If the resulting movement was not functional (as described above), the subject was excluded upon their own request.

For patients, not excluded at this point, the MeCFES parameters and setup were established. Then the ARAT was administered to establish the baseline score with the device off (natural score—AN_0_) and with the device active (score with the MeCFES—AM_0_). Finally, if the subject were still eligible and willing to participate, an intervention period was scheduled. In this fourth stage, individually prioritised tasks that involved the use of the tenodesis grip (primarily palmar prehension and pinch grip) were trained using the neuroprosthesis. At least 12 two-hour sessions, under supervision of an occupational therapist, were to be completed within two months. After the intervention period, the final evaluation with the ARAT was made with (AM_1_) and without (AN_1_) the device (stage 5).

Workshops were held before starting the study to train involved clinical staff in the ARAT administration, safety procedures, and set-up and working of the MeCFES. The study was approved by local medical ethics committees and the Ministry of Health.

### 2.4. Data Processing

We defined the following terms and change scores: an immediate effect (AM_0_-AN_0_), a therapeutic effect (AN_1_-AN_0_), a training effect (AM_1_-AM_0_), and a combined effect AM_1_-AN_0_. The immediate effect is the difference in ARAT score that the MeCFES induces at first application. The therapeutic effect is the change of ARAT from start to end of intervention, as obtained without stimulation. This would reveal a carry-over effect of the intervention. The training effect is the change in ARAT with the neuroprosthesis activated, from start to end of intervention period. This would indicate how much better or worse the subject would perform with the system, due to training. Finally, the combined effect is the comparison with the baseline before applying the neuroprosthesis to the end of training period with the neuroprosthesis and would indicate the combined result of training and assistive effect of stimulation that the system could induce. Primary outcomes were the median ARAT change scores and secondary outcomes were the proportions of subjects having positive change scores and the proportions exceeding the clinical relevant improvement.

Descriptive statistics were calculated as median and interquartile range (IQR [25%, 75%]) for the primary outcomes, age, time since onset of spinal lesion and the neurological level. The 95% confidence intervals (95% CI) were calculated for proportions the number of positive change scores and change scores that exceeded the clinically relevant level. Comparisons were made in MedCalc using nonparametric statistics (Wilcoxon signed-rank test). Levels were ranked (C5, C6, and C7) for the statistical analysis. Age, time since onset and neurologic level were correlated with the primary outcomes using the Spearman rank correlation (*ρ*). In a post hoc analysis, the Kruskal-Wallis (or Mann-Whitney  *U* test), was used to test differences in the various stages with respect to age, time since onset, level, sex and spinal unit.

## 3. Results

### 3.1. Participants

Screening the spinal unit databases for patients admitted for the last 8 years resulted in *N*_0_ = 253 records of cervical spinal cord level with complete anamnesis and contact information. Both units had similar (*P* = 0.2) number of cases, 131 versus 122. The median age was 40 (IQR [31; 54]), time since onset was 8 years (IQR [5; 14]), and 213 (84%) of the screening population were males. After applying the level, age, and health status exclusion criteria, *N*_1_ = 169 records with C5–C7 level of lesion were considered for physical examination in stage 2 (see Figure 1). These subjects were contacted and asked to participate in the study and *N*_2_ = 79 subjects (95% CI [25%; 37%]) were willing and able to undergo the clinical examination in stage 3.

The clinical examination excluded further 50 subjects due to lack of motor response to the stimulation, nonfunctional hands, and active hands that could not be increased by MeCFES or refusal to continue (see Table 1 for details). The dominant hand was used in all cases (20 right-handers).

### 3.2. Intervention

Thus 29 subjects were enrolled for MeCFES assisted occupational therapy programme, training the prioritized ADL for two hours under guidance of the occupational therapists. A plurality of activities were prioritized. These in order of occurrence were writing, dressing, dining, cooking, manipulating small or heavy objects, putting books on shelves, and so forth. Also special activities like personal hygiene and, for example, loading the wheelchair into their car, were trained. All subjects except two drop-outs (a surgical intervention and a botulinum toxin treatment) completed the intervention period, training ADL with the MeCFES. Thus final measurements were taken on *N*_4_ = 27 compliant patients equalling to 11% (95% CI [7%, 15%]) of the cohort. The ARAT scores from the four conditions are listed in Table 2.

### 3.3. Primary Outcomes

**Figure 2:**
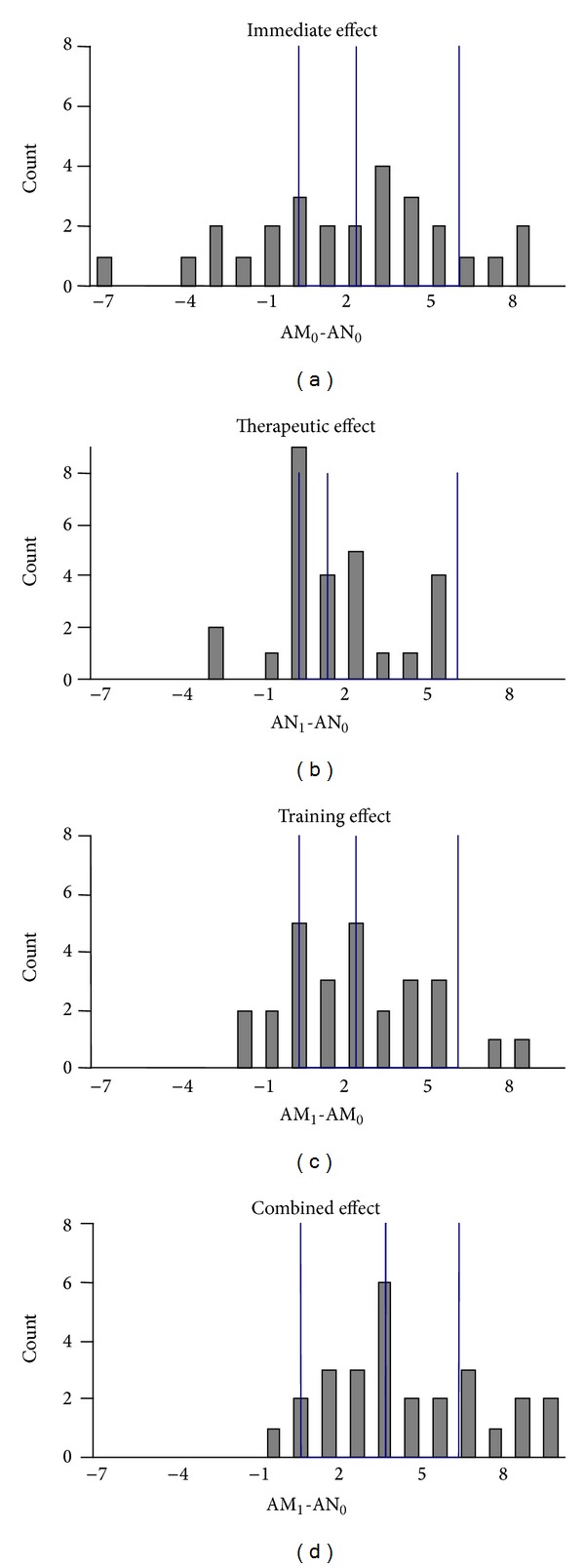
Histogram plots for the change scores with lines at zero, median, and 5.7 points limits.

Figure 2 shows the histogram plots for the distribution of the change scores of the immediate, therapeutic, training, and the combined effects.

We found a significant immediate effect (AM_0_-AN_0_) increasing the hand function in 63% of the compliant subjects, of whom 15% exceeded the clinically relevant change of at least 5.7 ARAT points. Though less pronounced, we also found a significant therapeutic effect (AN_1_-AN_0_), which means that 56% of the subjects improved the hand function as a carry-over effect from the MeCFES intervention period. However, this effect was small and none of the subjects exceeded the clinical relevant threshold.

As for the training effect (AM_1_-AM_0_), we found a highly significant increase of 2 points. Thus training had increased the ARAT score in 67% of the participants and in 11%, this change was clinically important.

The combined effect with training (AM_1_-AN_0_) yielded a highly significant median increase of 4 (IQR [2.3, 4.8]) points of the test score yielding 89% of enrolled patients or 9% (95% CI [6%, 14%]) of the screening sample (*N*_0_). Of these, eight (30%) exceeded the clinically relevant change. See Table 3.

Some subjects decided to complete the protocol though not having an immediate improvement of the ARAT score, because they experienced improved grasp on specific tasks. These subjects actually improved during the intervention period and the change was attributable to items of the ARAT requiring strength (grasp and pouring).

### 3.4. PostHoc Analysis

We investigated possible relations between baseline natural ARAT (AN_0_) score and change scores. Correlations with immediate, therapeutic, or combined effects were poor and nonsignificant. A negative correlation (*ρ* = −0.5, *P* = 0.006) of the training effect with baseline score was found. Ranked level of lesion and immediate effect (AM_0_-AN_0_) were positively correlated (*ρ* = 0.5, *P* = 0.005). However, the other change scores were non-significantly correlated with level.

No significant associations between sex or time since onsetand selection process or primary outcomes (immediate-, therapeutic-, training- and combined effect of MeCFES) were found. However, there was an association between spinal unit and the immediate effect (*P* = 0.003). Subjects with initially negative immediate effect were all from the same spinal unit and this unit had larger training effects (*P* < 0.03). Small non-significant correlations were found with respect to age and time since lesion in all change scores. The selection process (stage 1 to 3) had a significant trend (*P* < 0.01) towards examining subjects of a lower age from the cohort, but subjects completing the protocol (stage 5) were not significantly different (*P* = 0.47) from the examined group (stage 3). No accidents or adverse effects related to the MeCFES use were experienced.

## 4. Discussion

Clinical relevance of an assistive technology is influenced by the function it offers and the amount of potential candidates as well as the possibility to access these candidates in a cost effective way. By screening electronic databases about a third could be excluded. Another third were excluded mostly due to logistic problems when making contact. It can be questioned if some of these would be candidates for a neuroprosthesis, thus presenting a conservative bias of the final estimate. The more resource consuming process was the clinical evaluation involving the last third of the cohort.

### 4.1. Limitations

Electrode location was time consuming and subject to individual variations. In general, it was possible to adjust the coordination of thumb and finger flexors by modifying the placement of one or both electrodes and hence to some extent control the individual activation of thumb and fingers, permitting a functional grasp. The possibility to thus control multiple muscle activations by a single channel has practical advantages when placing electrodes. However, from a technical standpoint it has been clinically observed that it would be better to employ more channels for selective muscle stimulation.

In this study, only one of the hands was included. It was however observed and tested during the study that at least two of the compliant subjects could benefit from bilateral systems. Bilateral application merits further investigation.

There was a significant difference at baseline between the spinal units with respect to recruiting subjects for the intervention period. This may imply that one unit was more conservative when enrolling patients who did not show immediate benefit of the neuroprosthesis, hence potentially not including subjects that could benefit from training.

Being a prototype, mounting electrodes and adjusting system parameters had to be taken care of by well-trained clinicians. However, one subject was able to don the system by himself after some training. Some technical problems were encountered with establishing correct electrode contact and positioning. This as well as the difficulty of the iterative MeCFES parameter adjustment was resulting in suboptimal application in some cases. Wires connecting electrodes with the electronic device were prone to breakages and tangles. This also caused some inconveniences.

We found no significant differences of sex, age, time since onset and, level with respect to other studies [1, 13, 30, 31] and we therefore believe that our sample is representative for the spinal cord population. Our estimate of potential candidates is also in line with the findings of Gorman et al. [13] and may thus be realistic, if not conservative.

### 4.2. Implications

A safe method of improving hand function has been demonstrated. Eventually a less cumbersome implementation of the myoelectric controlled FES is imperative for allowing full benefits for the patient.

To test compliance with activities of daily living, the neuroprosthesis was tested by the subjects for a period in an occupational therapy setting. During this period, patients and therapists found that the neuroprosthesis allowed the execution of new tasks that required more grasp force.

Surprisingly a therapeutic effect was discovered showing that the intervention may provide functional recovery. It should be addressed specifically in further studies, whether it is because the neuroprosthesis permitted exploring new grasp strategies, due to neuromuscular changes [32] induced by the stimulation [33] or simply a training effect of occupational therapy. This should be confirmed in a controlled study.

Most likely candidates have C6/7 level of lesion, but also people with C5 may benefit even though the immediate effect was lesser than for C6/7 level subjects. An immediate improvement of hand function seems a good starting point for deciding whether to employ the method and taking into account that training may increase the effect. The negative correlation between initial ARAT score and the effect of training may imply that less functional hands are more susceptible to training. An explanation could be that the encouraged use of the hands strengthens atrophied muscles in the otherwise nonused hand. Further studies should investigate dose-response effect to establish optimal application duration. Another issue to address is if the training has to be supervised by an occupational therapist or if it could be considered as a habituation period during home use. In general, the patients reported that the MeCFES was useful for practical activities, even in cases where it was not evident from the ARAT change score. We will analyse this finding further.

In conclusion, we found that a noninvasive neuroprosthesis has demonstrated that it can enhance the tenodesis grip of subjects with a cervical spinal cord lesion. The device uses myoelectric signals from wrist extension to control neuromuscular stimulation of the finger flexors. Approximately 9% of the cervical spinal cord population may be candidates for the technique. The majority of ineligible subjects (2/3 of the cohort) were excluded by screening of clinical records and telephonic contact. The remaining ineligible patients were excluded after a clinical examination.

Long-term testing in an occupational therapy setting demonstrated that the method is useful for activities of daily living and a clinically relevant improvement of the hand function is documented through hand function testing.

Besides the immediate effect, there might be some therapeutic benefits to investigate employing this kind of neuroprosthesis in the occupational therapy.

## Figures and Tables

**Table 1:** Reasons for exclusion in the three first stages of selection.

Exclusion in stage 1		Exclusion in stage 2		Exclusion by examination	
By level	70	By health problems	5	No innervation	24
By age	3	No functional hand	10	No functional hand	11
Death/disease	11	Near normal hands	10	No gain by stimulation	9
		Would not participate	22	Could/would not participate	6
		Logistic problems	43		

Total (*Z*_1_)	84	Total (*Z*_2_)	90	Total (*Z*_3_)	50

**Table 2:** ARAT scores sorted by spinal unit (UN/US) then neurological level ASIA scale and AIS (ASIA impairment scale). Scores with the MeCFES off (AN_0/1_) and with the MeCFES active (AM_0/1_) before (0) and after (1) the intervention period. Data are summarized as median values with interquartile ranges (IQR). Clinically relevant changes exceeding 5.7 points are marked^§^.

Patient	Level	AIS	AN_0_	AM_0_	AN_1_	AM_1_
UN(1)	C5	A	15	11	14	15
UN(2)	C5	A	16	15	19	18
UN(3)	C5	A	26	19	23	27
UN(4)	C5	A	31	31	32	33
UN(5)	C5	A	32	29	37	33
UN(6)	C5	A	33	33	35	33^§^
UN(7)	C5	A	31	35	36	37
UN(8)	C5	B	18	18	20	23^§^
UN(9)	C6	A	14	13	19	20
UN(10)	C6	A	24	21	21	23
UN(11)	C6	A	27	28	29	30
UN(12)	C6	B	18	16	20	20
UN(13)	C6	B	25	32^§^	25	34^§^
UN(14)	C6	B	26	31	27	29
UN(15)	C6	C	40	41	40	44
UN(16)	C7	B	28	30	33	35^§^
UN(17)	C7	B	34	36	38	37
US(18)	C6	A	25	28	26	28
US(19)	C6	A	28	31	28	36^§^
US(20)	C6	A	19	27^§^	20	28^§^
US(21)	C6	A	32	37	32	38^§^
US(22)	C6	A	36	40	36	39
US(23)	C6	A	38	44^§^	38	43
US(24)	C6	A	40	43	40	43
US(25)	C7	A	45	48	47	46
US(26)	C7	A	25	29	25	29
US(27)	C7	A	41	49^§^	41	49^§^

Median			28	31	29	33
IQR			[25; 34]	[24; 37]	[22; 37]	[28; 38]

**Table 3:** Summary of change scores of the 27 patients completing the intervention period: immediate (AM_0_-AN_0_), therapeutic (AN_1_-AN_0_), training (AM_1_-AM_0_), and combined effect (AM_1_-AN_0_) with interquartile range. Number of positive change scores and number of clinically relevant improvements are listed in the last two rows together with their 95% confidence intervals.

Change score	AM_0_-AN_0_	AN_1_-AN_0_	AM_1_-AM_0_	AM_1_-AN_0_
Median [IQR]	2 [−1,4]	1 [0,2]	2 [0,4]	4 [2,6]
(Wilcoxon—*P*)	(*P* < 0.02)	(*P* < 0.01)	(*P* < 0.0001)	(*P* < 0.0001)
Count of positive change scores	17	15	18	24
95% CI	[44%, 78%]	[37%, 72%]	[48%, 81%]	[72%, 96%]
Count exceeding 5.7 points	4	0	2	8
95% CI	[6%, 33%]	[0%, 13%]	[4%, 28%]	[16%, 49%]
